# A fructosylated peptide derived from a collagen II T cell epitope for long-term treatment of arthritis (FIA-CIA) in mice

**DOI:** 10.1038/s41598-021-95193-2

**Published:** 2021-08-30

**Authors:** Clara Wenhart, Hans-Peter Holthoff, Andreas Reimann, Zhongmin Li, Julia Faßbender, Martin Ungerer

**Affiliations:** 1 Advancecor, 82152 Planegg-Martinsried, Germany; 2 ISAR Bioscience, Semmelweisstr. 5, 82152 Planegg-Martinsried, Germany

**Keywords:** Antigen processing and presentation, Immunology, Autoimmunity

## Abstract

Rheumatoid arthritis (RA) is a systemic inflammatory autoimmune disease which affects primarily the joints. Peptides of several proteins have shown an effect in some experimental animal models of RA. We investigated arthritis development in male DBA/1 mice which were injected with bovine collagen II (bCII) and human fibrinogen (hFib) on days 0 and 21, leading to stable and reproducible disease induction in 100% of immunized mice (FIA-CIA). In a second study, two bCII—derived peptides were given three times in the course of 6 weeks after FIA-CIA induction to test for impact on arthritis. Mice were scored weekly for arthritis and anti-citrullinated peptide antibodies (ACPAs) were determined in the sera taken on days 0, 14, 35, 56 and 84. Histology of the hind paws was performed at the end of the experiment. Intravenous administration of peptide 90578, a novel fructosylated peptide derived from the immunodominant T cell epitope of bCII, at a dosage of 1 mg/kg resulted in significant beneficial effects on clinical outcome parameters and on the arthritis histology scores which was sustained over 12 weeks. Survival tended to be improved in peptide 90578-treated mice. Intravenous administration of pure soluble peptide 90578 without adjuvants is a promising approach to treat RA, with treatment starting at a time when ACPAs are already present. The results complement existing data on peptide “vaccination” of healthy animals, or on treatment using recombinant peptide expressing virus or complex biological compounds.

## Introduction

Rheumatoid Arthritis (RA) is one of the most common systemic autoimmune diseases with a prevalence of 0.5–1% in the overall population^1^. Several risk factors contribute to the development of RA, including genetic factors, age and gender, smoking, socioeconomic status and infections^2^. Pathophysiology includes inflammatory arthritis, but also extraarticular manifestations of disease with cardiovascular, pulmonary, psychological and skeletal disorders^3^. Rheumatoid factor (RF), anti-citrullinated peptide antibodies (ACPAs)^4^ and anti-collagen-II antibodies^5^ are commonly found in sera of patients with RA.

Therapy of rheumatoid arthritis mainly relies on disease modifying anti-rheumatic drugs (DMARDs) such as the conventional synthetic methotrexate, biological drugs (TNF-, T cell, B cell or IL-6R-inhibitors) or the targeted synthetic janus kinase inhibitor tofacitinib. Although there are many therapeutic approaches, remission or low disease activity is not achieved in all patients, or the patients lose responsiveness over time^6^. In addition, some of those therapeutics result in nonspecific immunosuppression, which can lead to complications such as infections or cancer^7–9^. Thus, further study of RA and search for therapies is needed.

Peptides can regulate the immune response to induce tolerance of certain antigens and are a promising approach in mitigating or even curing autoimmune diseases and other immunological disorders^10,11^. The immunodominant T cell epitope of bovine collagen II (bCII) has been shown to be a peptide with only eight amino acids at positions 260–267 of the collagen α-chain^12,13^. Several studies which investigated the effects of this peptide on the outcome of collagen-induced arthritis (CIA) in mice have been conducted and evaluated. Coimmunization with CII and of CII-derived peptide 245–270 [s260,261,263] leads to a dose-dependent effect on the incidence of arthritis in the mouse CIA model in DBA/1 mice. When the peptide was mixed together with collagen at a dose which exceeded the amount of collagen II molecules by 480-fold on a molar basis, this mixture succeeded in completely preventing the development of arthritis in CIA mice^14^. Intravenous or intranasal administration of a galactosylated CII 259–273 in complex with MHC-II-molecules A^q^ reduced the incidence and severity of CIA in B10.Q mice and ameliorated chronic relapsing disease^15^. Gene therapy with hematopoietic stem cells which had been infected with lentiviral particles expressing the CII 259–270 peptide on MHC-A^q^-complex several weeks before the immunization reduced the rate and the severity of CIA in DBA/1 mice^16^. In a rat model of adjuvant-induced arthritis (AIA), s.c. injections of a multi-epitope peptide reduced disease severity^17^.

With the exception of the multi-epitope peptide tested in AIA rats^17^, these peptide administrations relied on cofactors or recombinant viral gene transfer^15,16^ or were only effective if given before or simultaneously with the immunization^14,16^. Since we wanted to study a disease model which should be closer to the condition of human RA patients, we investigated the administration of peptides derived from the immunodominant epitope of CII in CIA without an additional MHC complex in a long-term mouse model of RA when they were given after the induction of arthritis. We investigated the effects of a novel fructosylated modification of the epitope peptide and compared them to those of the non-fructosylated peptide variant.

First, we established a mouse model of combined human fibrinogen (hFib)/bCII injection in our laboratory. The CIA mouse model has long been established in rheumatoid arthritis research and is well described^12,18–20^. However, there are contradictory reports on the formation of anti-citrullinated protein antibodies (ACPAs) in this model^21–24^. ACPAs, commonly detected by anti-cyclic citrullinated peptide antibody (anti-CCP)-ELISAs are the most important diagnostic serologic factor of human RA and are predictive for the severity of the disease^25,26^. To better reproduce the features of human RA in the mice, we used a modified immunization method based on the publication by Ho et al.^27^ with a combined bCII/hFib basal injection and hFib boosting (referred to as FIA-CIA). Here, we report the resulting stable disease model which developed in 100% of treated mice, and which is characterized by anti-citrullinated-fibrinogen antibodies. We then investigated the effects of the fructosylated and non-fructosylated bCII-derived peptides on arthritis development which were given three times in the course of six weeks after establishment of the disease phenotype.

## Materials and methods

### Animals

Male DBA/1 mice were purchased from Janvier (Janvier Labs, France). The mice were housed under standard housing conditions in individual ventilated cages (IVC) and were fed a high-fat diet (JL Maus 5K20mod.; ssniff Spezialdiäten GmbH, Soest). To minimize the risk of fighting, mice were housed in groups of three per cage. Enrichment material contained nestlets (NES3600-NESTLETS, Ancare, Bellmore, NY), other nesting material (Bed r´Nest, The Andresons, Maumee, Ohio) and cellulose (Covetrus). After arthritis onset the litter was upped so that the mice could reach food and water standing on four paws. Mice were 8 weeks of age at the time of the first immunization. A total of 47 mice were used in the experiments. In the pilot study, 10 mice were immunized according to the protocol which is outlined below and 2 mice served as native controls without immunization. For acclimatization, mice were obtained at an age of 6 weeks and housed for 2 weeks before the start of the experiment. In the therapy study, 30 mice were immunized and randomly distributed to the three treatment groups. 5 mice were investigated in the Complete Freund´s Adjuvant (CFA) control group, in which no therapeutic peptide was added. Sample size calculation was done a priori based on other RA mouse model effect sizes and a power of 80%.

Personnel which handled or investigated these mice were blinded to the treatment. All animal experiments were performed in accordance to Directive 2010/63/EU and approved of by the Government of Upper Bavaria, reference number: 55.2Vet-2532.Vet_02-17-94, based on prior evaluation of animal study plan design and group sizes by the certified bio-statistician Dr. Peter Klein. The description of all procedures involving animals was done according to the ARRIVE Guidelines in reporting in vivo experiments^28^.

### Immunization

Bovine collagen II (bCII; Chondrex, Inc.) and human fibrinogen (hFib; Millipore/Calbiochem) were dissolved according to the manufacturers´ instructions to a final concentration of 4 mg/ml for bCII and 8 mg/ml for hFib. Then each solution was emulgated separately in a 1:1 ratio with complete Freund´s adjuvant (CFA; Chondrex, Inc.) containing 2 mg/ml *M. tuberculosis* H-37 RA. For emulgation, two luer lock glass syringes were linked via a micro-emulsifying needle (Sigma-Aldrich) and the substances were mixed until there was a stabile emulsion.

Mice were anesthesized with isoflurane (CP-Pharma) at a concentration of 1.5–2%. For analgesia, Novaminsulfon (metamizol; bela-pharm) was given subcutaneously (s.c.). Then 50 µl of bCII-CFA emulsion was administered s.c. at the base of the tail and 50 µl of hFib-CFA emulsion was administered s.c. at the left flank. On day 21 after the first immunization, mice were boosted with 50 µl hFib in incomplete Freund´s Adjuvant (IFA) s.c. at the left flank. CFA control mice were administered the equivalent doses of CFA and IFA emulgated with 0.9% NaCl to reach equal injection volumes. Native control mice did not receive any immunization. Both CFA and native control mice served as negative control in the experiments.

For treatment of pain in arthritic animals, Metacam (meloxicam; Boehringer Ingelheim) was used at a dosage of 1 mg/kg. For treatment of severe local reactions to the first immunization, afflicted animals were treated with 10 mg/kg Baytril (enrofloxacin; Bayer) for 5 days per os.

### Clinical investigation

Paws were measured weekly. For that purpose, the mice were anesthesized shortly with isoflurane and the carpal and tarsal joints were measured via a caliper (Käfer Messuhren, Germany)^19^. In addition, each paw was scored according to an arthritis scoring system (score 0 = normal paw; score 1 = 1 toe inflamed and swollen; score 2 =  > 1 toe swollen or carpal/tarsal joint swollen; score 3 = entire paw inflamed and swollen; score 4 = very inflamed and swollen paw or ankylosis of the paw). Scores were added up to a total score of 0–16 per mouse. Upon development of arthritis, mice were examined daily. If an animal did not put weight on or use a paw anymore, it was euthanized immediately.

### Synthesis of peptides

**Figure 1:**
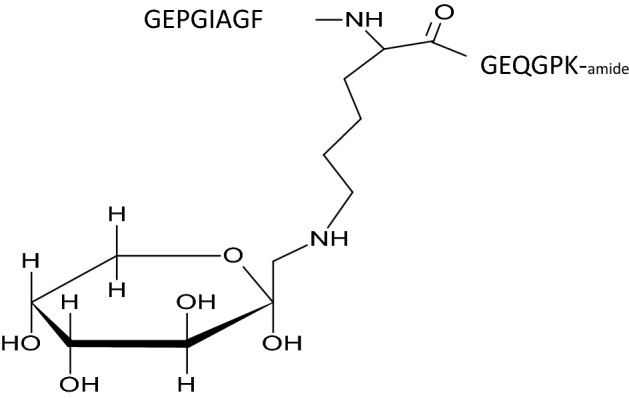
Novel fructosyl structure of peptide 90578. The peptide structure was modified by adding a cyclic fructosyl structure to the lysine residue. To enhance legibility, this lysine residue is shown as chemical structure, whereas the other amino acids of the peptide are just indicated as single letter codes.

Peptides represented the immunodominant T-cell determinant of bCII, peptides 256–270 of the α-chain^12^. Peptide 90091 was synthesized with the amino sequence GEPGIAGFKGEQGPK. Peptide 90578 was based on the same amino acid sequence, but posttranslationally modified by adding a fructosyl residue to the lysine at position 264, thus creating a novel chemical structure core (see Fig. 1). Both were synthesized by Biosyntan Berlin according to described protocols of fluorenylmethoxycarbonyl (FMOC) resin-based amino acid chain elongation. Both peptides were purified up to 95% (< = 95%) by means of HPLC and analyzed by MALDI-TOF mass spectrometry.

### Serum collection and peptide treatment application

Serum was collected on days 0, 14, 35 and 56 of the experiment via bleeding of the tail vein. Treatment with peptides 90091, 90578 (at a concentration of 1 mg/kg body weight) or 0.9% NaCl was applied intravenously on day 14, 35 and 56. Mice were randomly distributed to the three treatment groups. Treatment compounds were numbered 1, 2 and 3. To minimize confounders, each of the three mice per cage received a different treatment. All clinical and histological investigators were blinded to the treatment until data analysis was finished. For comparability between the therapy groups, all mice, including the CFA control mice, received a daily dose of 1 mg/kg Metacam (meloxicam) from day 14 after the first immunization on until the end of the experiment. Mice were killed on day 84 of the experiments via intracardiac punction and blood withdrawal under ketamine/xylazine anaesthesia.

### Measurement of mouse antibodies by ELISA

ELISA plates were coated with 100 µl per well of either 1 µg/ml bCII (Chondrex, Inc.) or 1 µg/ml hFib (Millipore/Calbiochem) or 1 µg/ml citrullinated hFib (Cayman) or CCP peptide at a concentration of 10 µg/ml in coating solution for 1 h. The CCP peptide we used for the selected CCP assay had the following sequence: HQCHQEST(cit)GRSRGRCGRSGS, with disulphide bonds spanning C3 –C16.

All subsequent procedures were performed at room temperature (RT) and incubations were done on a microtiter plate shaker. The coated plates were washed three times with PBST (PBS, 0.1% Tween-20), blocked with 100 µl/ well of blocking solution (PBST, 1% BSA) for 1 h, and washed again. Blood serum samples from mice were diluted 1:100 in PBST + 1% BSA and 100 µl were transferred to the blocked ELISA pates and incubated 2 h at RT. After washing with PBST, the ELISA plates were incubated with 100 µl/well of the detection anti-mouse antibody labelled with POD (Jackson Immunoresearch, #715–035-151, 1:10,000 dilution in PBST + 1% BSA), for 1 h. After washing, the POD was detected by incubation with 100 µl/ well of TMB substrate (Thermo Scientific, #34,029) until a maximal optical density (OD) of about 1 to 2 was reached. Finally, the colorimetric reaction was stopped with 100 µl/well stopping solution (1 M H_2_SO_4_) and the OD determined at a wavelength of 450 nm with a reference wavelength of 595 nm with the Tecan Infinite F 200 plate reader.

### Histology

Mice were examined post mortem for macro-anatomical pathology. Then, the right hind paws of the mice were amputated at the level immediately above the external malleolus. The skin was removed and the skinless paws were fixed in 4% paraformaldehyde at 4 °C overnight. Decalcification was performed in a mixture solution of 14% EDTA and 4% paraformaldehyde at a ratio of 1:1 for one day and subsequently in fresh 14% EDTA alone for 2 more weeks at RT under constant agitation. After this the paws were incubated overnight in a mixture medium of 30% sucrose dissolved in PBS and finally embedded in OCT compound (Tissue-Tek O.C.T. compound; VWR Chemicals, Leuven, Belgium).

Frozen sagittal sections (7 µm) were cut in series with an interval of 500 µm using a cryostat (temperature, − 19 °C; Leica Biosystems, Nussloch, Germany, and Buffalo Grove, IL) and mounted on Trubond microslides (Trubond 380, Catalog no: 63700-W1; Electron Microscopy Sciences; Hatfield, PA 19,440; USA). The sections were kept in a freezer at − 80 °C until use. There was a total of five sections in each paw to cover the distance from the external to internal malleolus.

Sections were stained with hematoxylin and eosin (HE) using standard protocols and were assessed for pathologic changes in the joints under the bright field illumination on a Zeiss upright microscope (Carl Zeiss AG, Oberkochen, Germany) by a blinded investigator.

A histological score was determined using a 0 to 3 scale based on the intensity and extent of change to the ankle and tarsal joints compared to native mice of the same age for each of five categories (score 0 = no changes; score 1 = slight change; score 2 = moderate change; score 3: marked change). The categories consisted of: inflammation, synovial alterations, cartilage degeneration, pannus formation and bony changes. The histological scores were expressed as the sum of the five categories for each section with a highest score of 15 per slide. The average of the scores of the middle three of five sections represents the histological score of one animal.

### Statistical evaluation

Results were compared by analysis of variance (ANOVA) after testing for normal distribution by Kolmogorov–Smirnov´s test using SPSS v19. If normal distribution was not present, Mann–Whitney test was used. Kaplan Meier plots were compared by log-rank testing.

### Ethics approval and consent to participate

All animal experiments were performed in accordance to Directive 2010/63/EU and approved by the Government of Upper Bavaria, reference number: 55.2Vet-2532.Vet_02-17-94.

## Results

### Immunization of DBA/1 mice with human Fibrinogen (hFib) and bovine Collagen (bCII) leads to arthritis in all immunized animals

In a pilot study, 10 DBA/1 mice were immunized according to the immunization scheme described above. Two additional mice which did not receive any immunization but were measured and scored according to the protocol served as native controls. Due to severe affection at the immunization sites, two of the immunized mice had to be euthanized soon after the first injection, and were excluded from analysis so that in the end, a total of 8 immunized mice and 2 native controls were used for evaluation of the experimental model.

**Figure 2:**
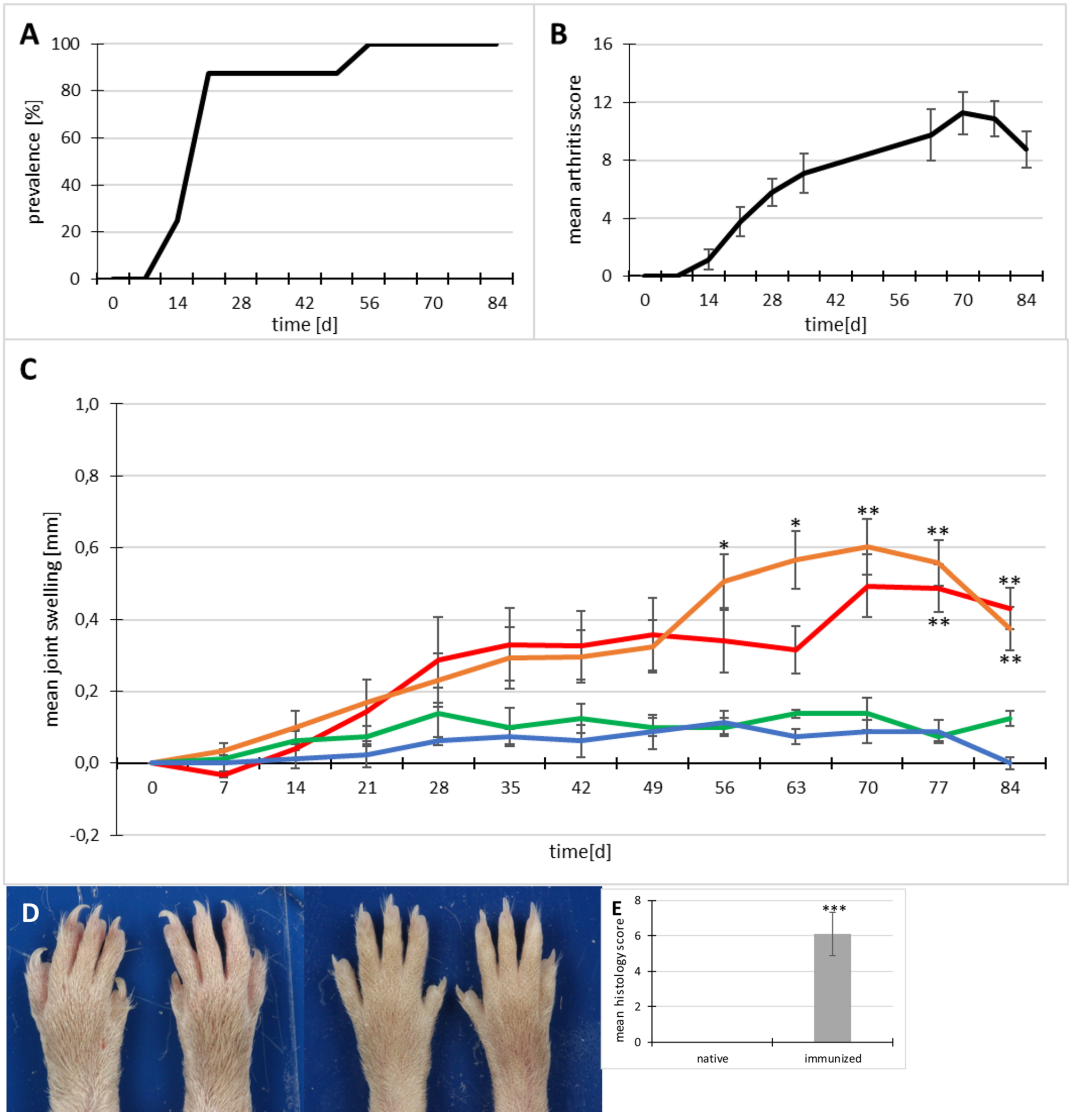
Clinical and histological results of the pilot study for FIA-CIA. (**A**) Arthritis prevalence in immunized mice, (**B**) mean arthritis score of immunized mice, (**C**) mean swelling of joints of immunized (red: carpal; orange: tarsal) vs native control (green: carpal; blue: tarsal) mice, (**D**) Paws of immunized (left) and native (right) mice in comparison. Swelling and ankylosis of joints, toes are thickened and the claws prolonged in immunized mice. (**E**) Mean histology scores of immunized and native mice. Results for 8 immunized mice and 2 native controls are shown. Histology score of FIA-CIA mice was significantly increased compared to that of native (not immunized) controls: *p < 0.05, **p < 0.01, ***p = 0.002; error bars indicate standard errors of the mean (SEM).

As shown in Fig. 2A the mice began to develop signs of arthritis on day 14 of the experiment. On day 56 the prevalence of arthritis reached 100%. The arthritis score was also severe with a maximum mean score of 11.25 on day 70 (Fig. 2B). None of the native control mice developed clinical signs of arthritis.

Paw measurements via a caliper showed significant differences between immunized and native mice. (Fig. 2C). The measured joints, especially the tarsal joints, showed swelling starting on day 28, which was significantly increased between days 56 and 70 until the end of the experiment. These results occurred in parallel to the respective arthritis scores.

Pathology and histology confirmed the clinical findings. The paws showed severe inflammation and ankylosis on all limbs. Figure 2D shows the marked difference between the hind paws of immunized and native mice. The whole paws of the immunized mice showed severe swelling including tarsal joints and metatarsus. The digits were swollen and ankylosed and the claws were prolonged due to a lack of use and thus, less abrasion. The HE-stained sections of the right hind paws were investigated histologically. The mean histology score of the immunized animals ranged at 6.1 whereas the native mice showed no signs of arthritis (Fig. 2E). Main findings were inflammation, cartilage erosion, bone alterations and pannus formation. Representative histological images can be seen in Fig. 4 –CFA control and CFA + NaCl groups.

### The mice developed a strong antibody response to collagen and fibrinogen as well as citrullinated fibrinogen

For assessment of antibodies, various antigens were coated on ELISA plates, and the sera of the mice were added. Binding of antibodies to the antigens was then measured by adding anti-mouse antibodies labelled with POD.

**Figure 3:**
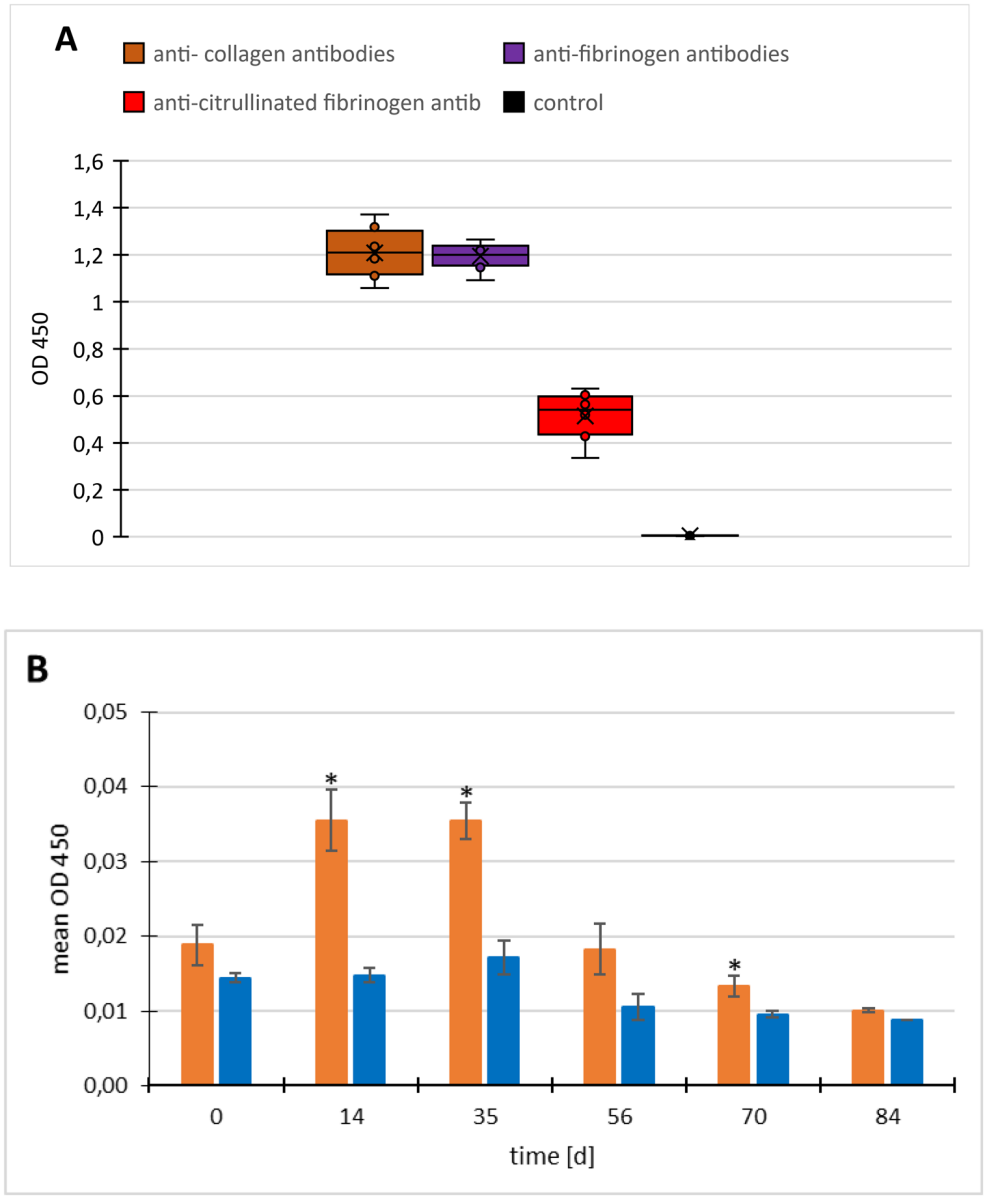
ELISA studies of the sera in FIA-CIA (**A**) Antibody titers against the immunizing antigens bCII (brown), hFib (violet) and citrullinated fibrinogen (dark red) on day 84, at the end of the experiment. Repeated measurements were carried out for all antibodies, and showed rapid increases of anti-fibrinogen, anti-collagen and anti-citrullinated fibrinogen antibodies to high values after 14–35 days, which did not change thereafter. For reasons of clarity we only show the values obtained at the final blood sampling upon sacrifice. Antibody titers of native control animals are shown in black, and were negative (zero values) for all 3 investigated antibodies. (**B**) Antibodies against CCP, orange: immunized; blue: native control; *p < 0.05, **p < 0.01; error bars indicate SEM. Repeated measurements are shown for anti-CCP antibodies due to their variation over time.

The mice developed a strong antibody response to the immunizing antigens bCII and hFib—the OD-450-values ranged between 1 and 1.4. The mice also developed an immune reaction to citrullinated fibrinogen, though to a lesser extent (OD about 0.5). The native control mice did not develop any of those antibodies (see Fig. 3A). Repeated measurements were carried out for all antibodies, and showed rapid increases of anti-fibrinogen, anti-collagen and anti-citrullinated fibrinogen antibodies to high values after 14–35 days, which did not change thereafter. For reasons of clarity we only show the values obtained at the final blood sampling at day 84 upon sacrifice.

We then tested the sera on ELISA plates that had been coated with one selected CCP peptide. Figure 3B shows that the antibody levels of the immunized animals reached low, but significantly higher levels than those of the native mice on days 14, 35 and 70. After that the OD values subsided to the same level as the native mice.

### Administration of peptide 90578 to immunized animals leads to reduced histological signs of arthritis

In a second experiment, FIA-CIA immunized mice were given either peptide 90091 or peptide 90578 at a concentration of 1 mg/kg i.v. into the tail vein on days 14, 35 and 56 after the first immunization. As positive controls, one group of mice received 0.9% NaCl as treatment. In addition, five mice were immunized with CFA/IFA alone at the two injection sites to serve as negative control animals (further referred to as CFA control mice). They did not receive any treatment.

Of the overall 30 FIA-CIA immunized mice, one had to be killed due to severe reaction at the injection site before treatment started and was excluded to the experiment. The remaining 29 mice were randomly distributed to the three treatment groups. Investigators were blinded to the treatments. Of those 29 mice, 6 had to be killed in the course of the experiment and were excluded to the analysis. For data analysis at the end of the experiment the remaining 23 mice were distributed as follows to the three treatment groups: 8 mice each received either peptide 90578 or 90091 and 7 mice received NaCl.

The following figures show data from the three different groups. Nearly all immunized animals developed arthritis, most starting between days 21 and 28.

**Figure 4:**
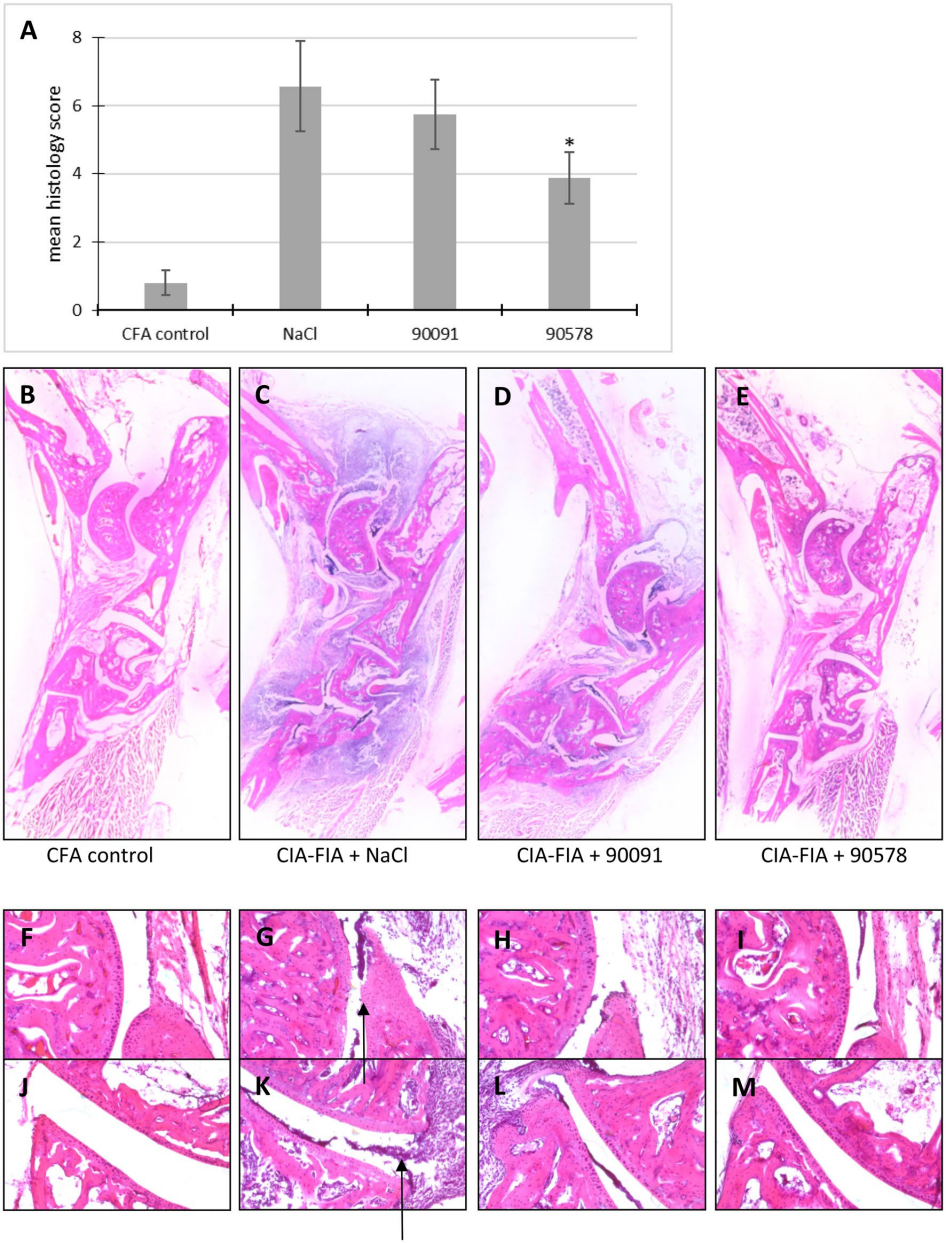
Histological findings in the therapy study with peptides 90091 and 90578 or NaCl. (**A**) Mean histology scores of CFA-immunized control mice and FIA-CIA mice treated with peptides 90091 (N = 8 independent animals), 90578 (N = 8) or NaCl (N = 7). Error bars indicate SEM; *p < 0.05 (all values compared to NaCl control mice). (**B**–**E**) Overview of the tarsus, (**F**–**I**) ankle joint, (**J**–**M**) tarsal joint. (**B**,**F**,**J**) Mice immunized with CFA alone, (**C**,**G**,**K**) FIA-CIA immunized mice treated with NaCl, (**D**,**H**,**L**) FIA-CIA immunized mice treated with peptide 90091, (**E**,**I**,**M**) FIA-CIA immunized mice treated with peptide 90578. Arrows indicate histological changes in the FIA-CIA group.

There was a significant decrease in the histology score of the 90578-treated group in comparison to the NaCl-treated group. The mice which had been given the peptide 90578 had an average histology score of 3.9, whereas the mice only treated with NaCl had a score of 6.6 (Fig. 4A).

Figure 4B–M show the differences between the HE-stained sections of the right hind paws. In the overview of the paws, the differences between CFA control animals and NaCl- and 90091-treated animals are apparent. The soft tissues around the ankle and metatarsus were severely enhanced in the NaCl-treated mice (Fig. 4C) and in the 90091-treated mice (Fig. 4D). In addition, the darker blue staining of the pictures of NaCl- and 90091-treated mice is due to a massive accumulation of lymphocytes in this region. In contrast to this, the 90578-treated mice showed less tissue enhancement and lymphocyte infiltration (Fig. 4E).

Ankle and tarsal joints were investigated in detail (Fig. 4F–M). The NaCl-treated mice showed signs of severe inflammation with lymphocyte infiltrates, cartilage and bone erosion. Pannus formation was also evident. 90091-treated animals had less lymphocyte infiltrates, but also pannus formation and cartilage erosion. In the 90578-treated animals, the cartilage was intact and there were only a few lymphocyte infiltrates in the subchondral bone. CFA-immunized control mice showed no significant joint alteration.

**Figure 5:**
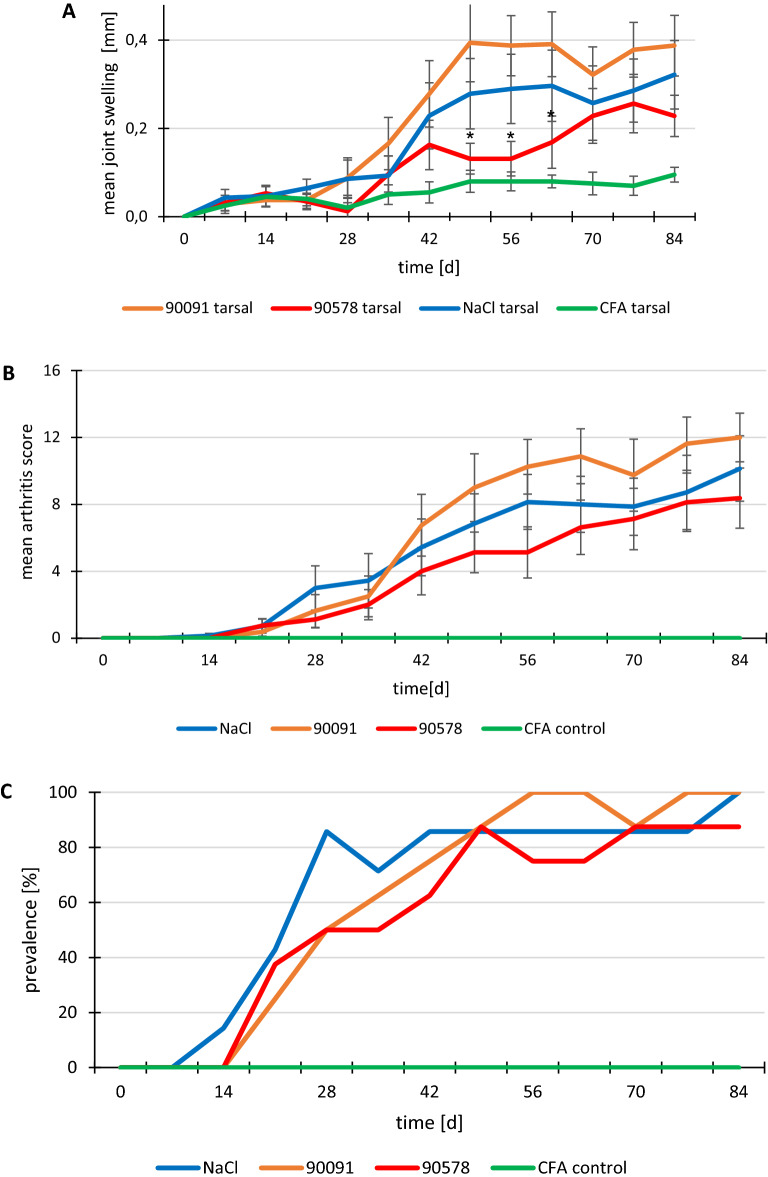
Clinical results of the therapy study with peptides 90091 and 90578 or NaCl. (**A**) Mean paw swelling of tarsal joints, (**B**) mean arthritis score, (**C**) prevalence of arthritis. Treatments: red: peptide 90578 (N = 8 independent animals); orange: peptide 90091 (N = 8); blue: NaCl (N = 7); green: CFA-immunized negative controls (N = 2); *p < 0.05 (all values compared to NaCl control mice); error bars indicate SEM.

Figure 5 shows that tarsal joints of mice receiving peptide 90578 had significantly lower swelling on days 49 and 56. The arthritis scores of the group treated with the peptide 90578 tended to be less severe than in mice treated with peptide 90091 or NaCl. Peptide 90091 even seemed to enhance arthritis, but not significantly. None of the CFA control mice showed any clinical signs of arthritis, paw measurements were significantly lower than in FIA-CIA immunized NaCl-treated mice.

**Figure 6:**
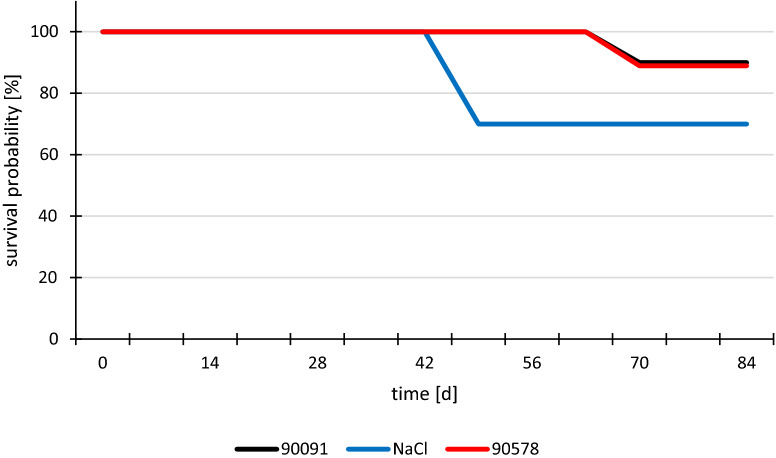
Kaplan–Meier-Chart of the survival probability in different treatment groups Mice were killed prior to the end of experiment when arthritis was too severe. Treatments: red: peptide 90578; black: peptide 90091; blue: NaCl.

Several animals had to be killed before the end of the experiment due to severe arthritis. As soon as an animal did not put weight on a paw anymore, it was euthanized immediately. The survival probability which is shown in the Kaplan–Meier chart in Fig. 6 shows the resulting survival rates of each group. In the NaCl-treated group, more and earlier drop outs were observed than in the groups that received peptide 90091 or 90578 as treatments. Statistical analysis based on log-rank testing did not reveal a significant difference between groups, which may be due to low number of cases. None of the CFA control mice had to be killed.

### Antibody levels of peptide 90091- and peptide 90578-treated animals were not affected by treatment

Although histological and clinical findings support an effectiveness of peptide 90578 in FIA-CIA mice, this effect could not be found in the antibody levels. In the ELISAs of the sera taken on days 35 and 84 there was no difference in anti-bCII or anti-hFib antibody titers between the three treatment groups. The antibody titers increased to the same levels regardless of treatment (data not shown).

## Discussion

In this paper we describe a mouse RA model in which the administration of a fructosylated peptide derived from bCII reduced clinical disease severity and damage to the joints in histological sections of the paws, when administered IV after disease had been established. These effects were not observed with the non-fructosylated homologous peptide.

The mouse model described here was modified based on the FIA-CIA model published by Ho et al.^27^, is highly reproducible and leads to severe arthritis of the paws in 100% of the animals. The mice established an immune reaction and developed antibodies directed to citrullinated fibrinogen as well as to the immunizing antigens. The presence of anti-citrullinated fibrinogen antibodies can be explained by two theories: One is that human fibrinogen gets citrullinated in the course of the preparation, so that the administered fibrinogen already contains citrulline residues, another theory is that the enzyme protein arginine deimidase 4 (PAD-4) that exists in polynuclear cells can citrullinate the peptides in the joints when those cells die and PAD-4 is set free^23^. Mass spectrometry showed that human fibrinogen (hFib) derived from Sigma-Aldrich or Calbiochem already contains certain citrulline residues^27^. However, the theory that PAD-4 can contribute to arthritis by citrullinating peptides and proteins cannot be dismissed. It has been shown that NETotic and necrotic granulocytes can citrullinate fibrinogen via PAD enzymes^29^. Upon immunization with PAD, some mice even developed antibodies against citrullinated fibrinogen^29^. Thus, the presence of anti-citrullinated fibrinogen antibodies is likely due to a mix of both those reasons.

Compared to the immunisation method described by Ho et al.^27^, we used only a tenth of the *Mycobacterium tuberculosis* dosage (0.05 mg instead of 0.5 mg per injection site) for the first immunization and half of the bCII dosage (0.1 mg per mouse compared to 0.2 mg). Despite the lower doses, we were able to induce the arthritis with sufficient severity in 100% of the immunized animals. The injection volume of only 50 µl per injection site also differs from the description of the CIA model in some publications. Most use an injection volume of 100 µl^19,31^ or a lower volume and instead a higher concentration of mycobacteria in the emulsion^18^. Here we present that in FIA-CIA, injection volumes of 50 µl and a *M.tuberculosis*-concentration of 2 mg/ml in CFA is enough to induce a severe arthritis. A reduction of *M.tuberculosis* in CFA incurs lower risks of severe ulcers or granulomas at the injection sites of the mice^31^ , which otherwise can lead to termination of the experiment before arthritis development and a need for more mice due to drop-outs.

Although the mice received a daily administration of the COX-2-blocker meloxicam upon development of arthritis, the severity of the arthritis increased even under this treatment. The dose was apparently not sufficient to have an unwanted impact on the joint inflammation, but visibly reduced the pain of the animals. Even the administration of meloxicam before the development of signs of arthritis in the therapy study did not prevent the onset of arthritis and the animals did not develop any adverse effects over the course of the experiment. Meloxicam has the advantage over other recommended analgesics in animal arthritis studies such as buprenorphin or paracetamol (acetaminophen)^30^ that it has to be given only once a day, is orally available and has less side effects. Thus, we propose it is possible to use meloxicam in the pain treatment of rheumatic animals in therapy studies. With regard to the 3Rs in laboratory animal welfare, reduction of the immunization dose and volume as well as application of pain killers should be considered in the refinement of mouse arthritis models.

Contrary to the findings of other groups^22,27^, we were not able to find relevant anti-CCP2 antibody levels, as determined with the commercially available assay in the sera of our mice. These tests showed no difference between immunized and native mice. Anti-CCP assays are used to detect the presence of ACPAs. As seen in the anti-citrullinated fibrinogen ELISA, our mice developed antibodies against at least one citrullinated protein. However, those antibodies could not be detected by the commercial anti-CCP2 tests. Also, using an assay with one single CCP coated on ELISA plates we found slight differences in antibody levels at three of six measurement days. Whether this result is due to a low antibody presence against citrullinated peptides in the sera or a difference in anti-CCP antibodies between mice and humans and thus, a low suitability of the tests for mouse studies remains to be elucidated.

The peptide 90578 had a significant effect on the histological outcome of the FIA-CIA in DBA/1 mice. When administered intravenously on three different time points after the first immunization, this peptide led to significantly reduced histological signs of arthritis in the treated animals compared to the control group. Peptides that mimic epitopes of collagen II have been shown to reduce or prevent the development of CIA in mice and rats, when administered before^32–35^ or in parallel to the immunization^14,36,37^, with an additional MHC-II molecule^15,16^ or by attaching the peptide to an immune or T cell binding peptide^38^. Some authors described oral or nasal administrations of peptides to induce mucosal tolerance^39–41^. But they also either applied the peptides before the induction of the arthritis^39,41^ or used additional immune modulators such as a cholera toxin- derived fusion protein^40^. Administration of peptides before the induction of arthritis does not replicate the situation found in human RA patients and this may be a critical point in finding new treatment strategies for RA. Also, the addition of molecules such as MHC or cholera toxin-derived proteins to the peptide increases the risk of severe adverse reactions which may be another reason why new treatment strategies subsequently failed in clinical studies in humans.

There are already some descriptions of CIA models in which the administration of modified peptides of collagen with two or three amino acid substitutions after the immunization or even after development of arthritis symptoms resulted in a therapeutic effect in rat^42^ or HLA-DR1 transgenic mice^43^. In a rat model of adjuvant-induced arthritis (AIA), s.c. injections of a multi-epitope peptide reduced disease severity^17^. This peptide also modulated T cell subsets in RA patients´ peripheral blood cells^44^. Also nanoparticles coated with peptides improved disease symptoms in HLA-DR4-IE-transgenic mice or depleted patient specific B cells^45,49^. Moreover, it has been shown that cyclic peptides can target RA patients´ ACPAs ex vivo^46^.

We now show that giving the fructosylated peptide 256–270 of bCII intravenously alone and two weeks after the induction of arthritis can lead to a long term effect in FIA-CIA in mice which is characterized by a significant reduction of histological signs of arthritis in mice. At the time of the first treatment application on day 14, the antibodies against bCII, hFib and citrullinated fibrinogen were already established, while arthritis was not yet prevalent. In human RA, development of autoantibodies against CCP often precedes the onset of arthritis for years^47,48^ and antibodies against collagen II are predictive for an early severe course of disease^5^. It would be desirable to intervene in the development of the disease so that symptoms can be attenuated or in the best case completely prevented.

An interesting aspect of our results is that the application of certain peptides or proteins works to switch the immune response to tolerance of certain antigens. There is some evidence that the application of peptides that mimic T cell epitopes can induce regulatory T cells that secrete anti-inflammatory cytokines such as IL-10 and TGF-β and thus lead to a suppression of antigen presenting cells^49^. The only difference between the two peptides in this study is the fructosylation of the lysin at position 264 of the 90578 peptide, whereas peptide 90091 is not modified. It has been shown that T cell recognition of collagen epitopes depends on glycosylation^15^ and native collagen II is also highly glycosylated^50^. The glycosylation of Lys 264 is oriented towards the T cell receptor (TCR) and has the potential to be a major regulator of T cell tolerance. It may be that the glycosylation at lysin 264 of the peptide 90578 leads to a better recognition by T cells and thus to the secretion of more anti-inflammatory cytokines such as IL-10, which then ameliorate the outcome of the arthritis in mice. Interestingly, glycosylation of the specific collagen epitope (aa 256–270) does not only play a role in CIA in mouse strains with the arthritis-susceptible A^q^ class II gene, but also in transgenic mice expressing human class II molecules associated with RA and in humans with RA^51^.

Also for the observed beneficial effects a reduction in T cell infiltration, differences in the migration of different subsets of T cells responding to or producing different cytokines may contribute. We will investigate these parameters in future studies. Further studies will also evaluate the reproducibility of the results in different mouse strains and other lab conditions.

## Conclusion

hCII-derived fructosylated peptide 90578 is a promising novel therapeutic approach to treat disease symptoms in FIA-CIA mice and potentially also in humans with RA. Further study of the compound is warranted.

## Abbreviations

ACPAs: Anti-citrullinated peptide antibodies
AIA: Adjuvant-induced arthritis
bCII: Bovine collagen II
BSA: Bovine serum albumin
CFA: Complete Freund´s adjuvant
CCP: Cyclic citrullinated peptides
DMARDs: Disease modifying anti-rheumatic drugs
FIA-CIA: BCII- and hFib-immunized mice which develop RA-like arthritis
FMOC: Fluorenylmethoxycarbonyl
hFib: Human fibrinogen
IFA: Incomplete Freund´s adjuvant
IL6R: Interleukin-6 receptor
MHC II: Major histocompatibility complex class II
PBS: Phosphate-buffered saline
RA: Rheumatoid arthritis
RF: Rheumatoid factor
TGF-β: Transforming growth factor beta
TNF: Tumour necrosis factor
